# Factors influencing the use of birth pools in the United Kingdom: Perspectives of women, midwives and medical staff

**DOI:** 10.1016/j.midw.2019.102554

**Published:** 2019-12

**Authors:** Sarah Milosevic, Sue Channon, Billie Hunter, Mary Nolan, Jacqueline Hughes, Christian Barlow, Rebecca Milton, Julia Sanders

**Affiliations:** a Centre for Trials Research, College of Biomedical and Life Sciences, Cardiff University, Neuadd Meirionnydd, Heath Park, Cardiff, Wales CF14 4YS, United Kingdom; b School of Healthcare Sciences, College of Biomedical and Life Sciences, Cardiff University, Eastgate House, 35-43 Newport Road, Cardiff, Wales CF24 0AB, United Kingdom; c College of Health, Life and Environmental Sciences, University of Worcester, Henwick Grove, Worcester, England WR2 6AJ, United Kingdom; d School of Healthcare Sciences, College of Biomedical and Life Sciences, Cardiff University, Heath Park Campus, Cardiff, Wales CF14 4XN, United Kingdom

**Keywords:** Water birth, Childbirth, Midwifery, Obstetric care, Qualitative research, Health services accessibility

## Abstract

**Objective:**

To identify factors influencing the use of birth pools.

**Design:**

Online discussion groups and semi-structured interviews, analysed thematically.

**Setting:**

United Kingdom.

**Participants:**

85 women and 21 midwives took part in online discussion groups; 14 medical staff participated in interviews.

**Findings:**

Factors influencing the use of birth pools were grouped into three overarching categories: resources, unit culture and guidelines, and staff endorsement. Resources encompassed pool availability, efficiency of pool use and availability of waterproof cardiotocograph equipment. Unit culture and guidelines related to eligibility criteria for pool use, medicalisation of birth and differences between midwifery-led and obstetric-led care. Staff endorsement encompassed attitudes towards pool use.

**Key conclusions:**

Accessibility of birth pools was often limited by eligibility criteria. While midwifery-led units were generally supportive of pool use, obstetric-led units were described as an over-medicalised environment in which pool use was restricted and relied on maternal request.

**Implications for practice:**

Midwives can improve women's access to birth pools by providing information antenatally and proactively offering this as an option in labour. Maternity units should work to implement evidence-based guidelines on pool use, increase pool availability (even where there appears to be low demand), and enhance awareness amongst medical staff of the benefits of water immersion.

## Introduction

United Kingdom (UK) guidelines recommend that women with uncomplicated labours should be offered water immersion analgesia (The National Institute for Health and Care Excellence, NICE, 2014). Many UK maternity units now provide birth pools and a low, but increasing proportion of women (Care Quality Commission, CQC, 2015) report using water immersion during labour (18%) or giving birth in water (10%) (CQC, 2019).

NICE (2014) recommend that women at low risk of intrapartum complications should be informed that they may choose to give birth at home, in a midwifery unit or in an obstetric unit. It is estimated that in England, 45% of women would be appropriate to commence labour under midwifery-led care (Sandall et al., 2014); however currently only 14% of births take place in midwifery-led units (Walsh et al., 2018), suggesting many women labour in obstetric-led settings despite a lack of a clinical indication. As the proportion of birth rooms with pools is lower in obstetric than in midwifery units (Which? Birth Choice, 2019), this is likely to affect women's access to intrapartum water immersion.

Immersion in water for labour provides a number of benefits, including analgesia (Eberhard et al., 2005; da Silva et al., 2009), relaxation (Benfield et al., 2010; Ulfsdottir et al., 2018), reduced likelihood of intervention (Burns et al., 2012; Henderson et al., 2014), increased breastfeeding initiation and higher maternal satisfaction (Lathrop et al., 2018). Limited research on the safety of waterbirth indicates no evidence of increased risk of an adverse outcome for women or neonates from immersion in water for labour or birth (Taylor et al., 2016; Shaw-Battista, 2017; Cluett et al., 2018; Vanderlaan et al., 2018).

Previous studies have found multifaceted barriers to the use of birth pools internationally, although little research has been conducted in the UK. Reviews of Australian policies and guidelines on pool use found them to be restrictive, focused on risk, and lacking an evidence base (Young and Kruske, 2013; Cooper, McCutcheon et al., 2017, in press; Cooper et al., 2018). While interventions such as epidural analgesia were found to be normalised and readily available, there were strict eligibility criteria for water immersion, which was presented in risk-based terms (Newnham et al., 2015, 2017). Despite clinical concerns relating to waterbirth (including neonatal water aspiration, infection and thermo-regulation) being commonly held (Young and Kruske, 2013; Nutter et al., 2014), it is argued that these are not substantiated by the available evidence (Young and Kruske, 2013).

Ineffective promotion of pool use is identified as a further barrier to uptake, with a reliance on women proactively requesting use of a pool (Russell, 2011, 2016). Exacerbating this, a lack of high-quality, evidence-based information for women on pool use for labour and birth has been highlighted (Young and Kruske, 2012; Nutter et al., 2014).

A shortage of skilled midwives to facilitate pool use (Russell, 2011, 2016), together with inexperienced senior staff (Plint and Davies, 2016), has impacted negatively on this practice. Cooper, Warland et al. (in press) report that inconsistent, prescriptive and sometimes unnecessary accreditation requirements prevent many practitioners in Australia from facilitating water immersion. Limited availability of water immersion facilities (Young and Kruske, 2012), along with physical concerns such as back problems for staff facilitating pool use (Nutter et al., 2014) are identified as further barriers.

Given that UK guidelines are supportive of water immersion in labour, it was important to conduct a UK based study to examine why this option is not being fully utilised in practice. As much previous research has focused on midwives’ experiences, it is suggested future studies should include the perspectives of other practitioners (Cooper, Warland et al., in press) and women themselves (Stark and Miller, 2009). This study aimed to identify factors influencing the use of birth pools in the UK, through exploring the attitudes and experiences of women, midwives and medical staff.

## Methods

This qualitative descriptive research was conducted as part of the larger POOL study, a cohort study investigating the safety of waterbirth for mothers and babies. This two-stage qualitative component of the research investigates factors influencing the use of birth pools in the UK. Stage one (described in this paper) comprises online discussion groups and semi-structured interviews. Findings will inform stage two; in-depth case studies of UK maternity units.

Separate online discussion groups were set up for midwives, women who were pregnant or had given birth within the previous year, neonatologists, obstetricians and paediatricians. The aim of the groups was to explore experiences, attitudes and beliefs in relation to the use of birth pools. This method was selected in order to engage geographically diverse participants, and to encourage open expression of views via an anonymous forum (Tates et al., 2009). An asynchronous approach was adopted, whereby participants could contribute to the discussion at a time convenient to them; a factor felt to be particularly important for new parents and clinical staff.

Due to difficulty recruiting medical staff to participate in online discussions, we offered brief semi-structured interviews to capture the views and experiences of this group. This method was selected in preference to a face-to-face discussion group as it would enable participation at a time and location convenient to individual participants, thus maximising recruitment. It is suggested that participants in individual interviews may generate a broader range of themes or ideas than those taking part in group discussions (Guest et al., 2017); an important consideration as participant numbers were anticipated to be low.

### Data collection

The discussion groups were open for 10 weeks from October to December 2018 and closed once no new themes were emerging. Each discussion started with an open question relating to access to pools for labour and birth, then follow-up questions were asked in response to topics raised by participants, who could contribute as much or as little as they wished. The groups were moderated during office hours by a qualitative health researcher (SM) and a registered midwife (BH), with the option for participants to report any posts they felt were offensive.

The semi-structured interviews were conducted face-to-face or over the telephone with obstetricians, neonatologists and paediatricians in January and February 2019. Interviews took between 8 and 16 minutes and were undertaken by SM. The interview guide comprised open questions relating to respondents’ experiences of waterbirth and perceptions of water immersion, including any perceived benefits or risks of pool use. Supplementary questions were then asked to further explore responses. All interviews were audio recorded with the permission of the participant, and transcribed verbatim.

### Recruitment

Recruitment was opportunistic for both parts of the study. The online discussion groups were advertised via networks including the Royal College of Midwives, Royal College of Obstetricians and Gynaecologists, British Association of Perinatal Medicine, NCT, Healthwise Wales and parenting forum Mumsnet. A link to the discussion group website was provided, where potential participants could view detailed information before deciding whether to take part. All participants were required to complete an online consent form and agree to comply with discussion group ground rules. They were asked to choose an anonymous public forum name and password, which they used to securely log in to the discussion.

The semi-structured interviews were advertised via professional networks, with adverts providing a brief overview of the interviews and a contact email address. Those who expressed an interest in the study were emailed a participant information sheet and given the opportunity to ask questions about the research. Consent was given verbally at the beginning of each interview and was audio recorded. Recruitment continued until data saturation was reached.

### Participants

Of 354 participants who registered to take part in an online discussion group, 106 (29.9%) contributed at least one post. Fourteen medical staff participated in interviews (see Table 1 for participant details).

**Table 1:** Discussion group and interview participants.

Group	N	Background	Data collection
Women	85	83.5% used a pool or bath in labour	Online discussion group
		63.3% had given birth in water	
Midwives	21	11 clinical midwives	Online discussion group
		5 midwifery managers	
		5 consultant midwives/clinical specialists	
Medical staff	14	7 consultant obstetricians	11 telephone interviews
		1 trainee obstetrician	1 face-to-face group discussion
		5 consultant neonatologists	
		1 consultant paediatrician	

All midwives and medical staff worked in NHS (National Health Service) settings in the UK.

### Ethical approval

The study was approved by Wales Research Ethics Committee 3. Informed consent was obtained from all participants.

### Data analysis

Verbatim interview and discussion group transcripts were uploaded to NVivo 11 and analysed thematically. Thematic analysis can facilitate the exploration of similarities and differences across large datasets (Braun and Clarke, 2006), and therefore was particularly useful in examining the variation within and between the participant groups of women, midwives and medical staff. An inductive approach was taken to allow the generation of unanticipated themes from the data. The six phases of thematic analysis proposed by Braun and Clarke (2006) were used to guide the analytic process (see Table 2).

**Table 2:** Phases of thematic analysis.

Analytic phase	Description
1	Discussion group and interview transcripts read several times and initial ideas of potential themes noted
2	Systematic coding of all data (conducted by SM)
3	Grouping of codes into possible themes and sub-themes
4	Themes reviewed to ensure accurate capture of coded data and reflection of dataset as a whole
5	Scope of each theme defined, and themes given concise names
6	Coherent narrative of themes constructed, supported by data extracts

To enhance reliability and validity of the analysis, once themes had been identified transcripts were independently coded by a second qualitative researcher (JH). The research team then discussed coding and interpretation of themes until consensus was reached.

## Findings

Women and midwives identified a range of factors influencing the use of birth pools. These could be grouped into three overarching categories, as outlined in Table 3: Resources, Unit culture and guidelines, and Staff endorsement.

**Table 3:** Factors influencing the use of birth pools identified by women and midwives.

Overarching category	Themes identified by women (W) and midwives (M)
Resources	Pool availability (W, M)
	Efficiency of pool use (W, M)
	Availability of waterproof cardiotocograph equipment (W, M)
Unit culture and guidelines	Eligibility criteria for pool use (W, M)
	Level of medicalisation of birth (M)
	Midwifery-led vs. obstetric-led care (W, M)
Staff endorsement	Midwife endorsement of pool use (W, M)
	Senior staff endorsement of pool use (W, M)
	Promotion of pool use (W)

### Resources

Availability of pools was commonly mentioned as a barrier to and facilitator of pool use, by women and midwives. A minority of participants stated that waterbirth was easily accessible in their locality due to sufficient pool availability. However, a lack of pools was frequently reported, meaning access was ‘on a first come, first served basis’ (W 412) or ‘a case of luck of the draw’ (W 166).

Midwives suggested a shortage of pools affected their ability to accommodate women's choices in labour and created a reluctance to offer waterbirth, in turn impacting on women's awareness and midwives’ experience of waterbirth.

*In units where there is only one pool, the number of clients who can use water as pain relief in labour is limited… This has an effect on staff encouragement to use. You wouldn't want to encourage use of something that may not be available. This then has a knock-on effect on staff frequency of using and thus confidence in it!!* (M 318)

Pool availability was a particular issue in obstetric-led units, which tended to have one or no pool, meaning women receiving obstetric-led care were generally unable to have a pool birth.

Women were aware of pool availability issues, causing anxiety for some. Participants described using strategies to ensure access to a pool, such as selecting a maternity unit with sufficient pools, contacting several units around their due date to assess occupancy, requesting a pool when telephoning the unit in labour, or planning a home birth. Women acknowledged that due to the cost, home waterbirth was not accessible for all.

Time taken for filling and cleaning pools between uses had prevented some women from being able to use a pool during labour. Inefficient allocation of birthing rooms was also highlighted, with instances of rooms with pools being occupied by women who did not wish to use a pool or those awaiting discharge.

*When planning my second delivery, I chose the unit based on how likely I was to get in the pool. The one I chose had the pool in a small internal room with no natural light; they were clear that it was only ever used for pool deliveries. The other potential unit, which was equidistant, stated that the pool room was the largest room they had and was a lovely environment, so it often got used for normal deliveries just because it was one of the nicest rooms. I was shocked that they would allow the pool to be blocked in this way.* (W 308)

Both women and midwives cited the lack of availability of waterproof cardiotocograph (CTG) equipment as restricting pool use.

### Unit culture and guidelines

In general, it was perceived as very difficult for women with risk factors to access water immersion. Hospital guidelines were viewed as rigid and arbitrary rather than evidence based. Women cited numerous reasons for not being allowed to use a pool, including need for CTG, being induced, being overdue, large baby, early rupture of membranes, gestational diabetes, intravenous drip, pyrexia, and meconium stained liquor. They also reported limits on when they could get into the pool, for example on reaching a specified level of cervical dilation. Others had been asked to leave the pool to give birth or during labour due to a lack of progress.

*I wasn't allowed into the pool… as my temperature was 37.5 and hospital policy said it needed to be 37.4. When I finally got my temperature to the ‘correct’ level (I took off all my clothes and opened all the windows) I was allowed in and my temperature immediately went down to normal levels.* (W 231)

*I was blocked* [from using a pool] *without genuine evidence based rationale – it was a blanket policy they have of not letting any woman with any level of GD* [gestational diabetes] *use the birth centre… Postcode lottery comes into play as other areas would let a GD mother use a pool, which tells me that the risk cited by some clinicians is debatable and not evidence based.* (W 202)

*They ran the pool for me at 7* *cms after my waters broke (last examination before this I was only 2* *cm so not allowed to get in yet!) and my daughter was born 35 minutes later. I had only been in the pool about 20* *mins.* (W 151)

Midwives agreed that unit guidelines could be restrictive and unsupportive of pool use. Some expressed concern that birth tended to be over-medicalised, particularly in obstetric-led units, leading to waterbirth being viewed as unusual and thus constrained.

*If midwife-led care were the default position… it may be that water would be viewed as a relevant normal form of pain relief for the most part. In the current climate “normality” is being viewed as something special, instead of usual.* (M 318)

The contrast between obstetric-led and midwifery-led care was also commonly highlighted by women. There was a lack of pools on obstetric-led units, and restrictions on pool use for women requiring monitoring. In contrast, low-risk women labouring in midwifery-led units (MLUs) generally described pool access as easy.

*If you have a very straightforward labour and are midwife led then it's relatively easy* [to access a pool]*… but for everyone else it's not.* (W 420)

[For my first baby] *I was not encouraged to have a water birth once transferred to the Consultant led unit… My second baby was born 4 months ago in a birthing pool of the same city hospital but on the MLU. It was a completely different experience. I got in the water once fully dilated… and gave birth in the water about 15 minutes later… I felt totally supported by the midwife present who helped me to have the birth I wanted.* (W 162)

### Staff endorsement

Women receiving midwifery-led care generally reported feeling supported to have a pool birth, both antenatally and during labour. However, a minority felt unsupported by midwives, noting some resistance to waterbirth.

[I] *opted to deliver on the MLU in the pool. Only 2 pools are available… However, I found the biggest barrier was the midwives. I was quickly made aware by overhearing talking outside my cubicle on the assessment unit that the midwives were not keen on doing a water birth. It took over an hour from me* [being] *assessed at 6* *cm and in agony with no pain relief to me getting in the pool whilst they waited for a willing midwife to come do my delivery.* (W 152)

Midwives themselves suggested staff resistance was due to a lack of confidence in supporting women to labour and give birth in water, arising from limited skills or experience.

*The frequency of waterbirths depends on… which staff* [are] *on shift. In my experience some midwives (a small minority) will find any reason to persuade a woman to get out of the pool. I think this is due to lack of confidence or maybe because they have had a bad experience in the pool.* (M 267)

Furthermore, clinically based midwives reported that a lack of support for pool use from obstetricians and senior midwives, notably where women had additional risk factors, had led to low confidence in promoting waterbirth. Particularly on obstetric units, senior staff were seen as not appreciating benefits of pool use, being resistant to change and promoting a medicalised approach.

*I worked in a unit that was not as comfortable with midwifery-led care in labour and consultants who did not think water immersion was something of value but only an added risk. When the Obstetric team and the matrons share these views it is difficult for band 5 and 6* [clinical] *midwives to shift the common practices and offer something different.* (M 242)

*I work on a consultant led unit and we have a birthing pool but you don't always get support from all the coordinators or Doctors to facilitate waterbirths. You very much feel “on your own” which means that if anything “goes wrong” it's on your shoulders… I am not that experienced in providing water births but would be more than happy to give it a go if I felt supported by seniors.* (M 256)

Women too noted a lack of support from obstetricians, highlighting that even where midwives are supportive of waterbirth they may have to advocate for this against directives from consultants.

*The midwives were led by the consultants and they were not confident to fight for my rights… The consultants were far too ready to implement interventions… They need educating that medicalising birth is not always the best way, then they won't bully the midwives.* (W 202)

Women emphasised a need for greater information about waterbirth antenatally. It was reported that information about waterbirth tended to be given only in response to questions, or during private antenatal classes, rather than being proactively raised by midwives as part of routine antenatal care.

*It was only when I did NCT antenatal classes (paid classes) that I understood the possible benefits of a birthing pool and could imagine me being in one. Had I not done those classes, I'm not sure if that information would have been available to me via the NHS and if that would have been something I wanted.* (W 154)

### Medical staffs’ views and experiences of pool use

The attitudes of medical staff towards pool use were predominantly described in terms of potential risks and benefits of water immersion.

### Perceived risks of pool use

Obstetricians and neonatologists/paediatricians (hereafter referred to as neonatologists to preserve anonymity) generally considered waterbirths were safe, with a small additional risk compared to births on dry land.

*I am prepared to agree that the absolute risk is probably quite low.* (O 1001)

*I think they are really probably only marginally less safe than dry land births.* (N 2001)

Despite this, several concerns were raised, including potential delay in recognition of intrapartum complications and delayed emergency treatment. Neonatologists considered water and meconium aspiration and infection due to unclean pool water to be risks, whilst obstetricians suggested waterbirth increased rates of severe perineal trauma and that prolonged water immersion results in perineal oedema complicating perineal repair. It was suggested maternal dehydration or over-hydration may be increased with pool use and that fetal monitoring is more difficult, potentially causing a delay in the detection of developing fetal hypoxia. Obstetricians identified health and safety risks of pool use for midwives, such as back problems due to bending and stretching to examine or support women during labour and birth.

Consistent with the rigorous eligibility criteria reported by women and midwives, medical staff proposed a number of factors they felt should contraindicate pool use (see Box 1).

Box 1 **Contraindications for pool use proposed by medical staff**

**Contraindications**  

• ‘High risk’ women
• Monitoring required
• Hypertension
• Intravenous access
• High BMI (body mass index)
• Gestational diabetes
• Infection
• Epilepsy
• Breech birth
• Large baby
• Pre-term birth
• Premature rupture of membranes
• First pregnancy
• Previous caesarean section

Alt-text: Unlabelled box

Interviewees acknowledged that their view of the safety of waterbirth was likely to be influenced by the fact they only attended births with complications.

*Undoubtedly what you'll find is neonatal people think very differently* [about waterbirth] *because… obviously we don't see all the uncomplicated* [births]*.* (N 2003)

*I think our view of* [waterbirth] *is a little bit skewed… just because we tend to get involved when they've gone wrong, so we… are not huge fans.* (O 1005)

Furthermore, medical staff highlighted the difficulty of establishing the relative risks of pool use, as it was not always clear whether complications were attributable to waterbirth or may have occurred in any case. It was identified that evidence on the safety of waterbirth is currently limited.

### Perceived benefits of pool use

Medical staff generally agreed pool use during labour was beneficial for the mother in terms of analgesia and relaxation, although some suggested there was limited evidence to support this.

[Pools] *are over hyped in terms of their pain relief.* (N 2001)

*I think there's quite good evidence that* [water] *helps with pain relief, not very strongly, but it's reasonable.* (O 1001)

Neonatologists generally considered there were no benefits of waterbirth for the baby and were concerned about potential harm. Obstetricians suggested pools provided effective analgesia for labour but were problematic for birth. There appeared to be some scepticism around the benefits of pool use, with some medical staff suggesting waterbirth was unnatural, pointing to the fact that other mammals give birth on dry land.

*I think the idea of waterbirth is mis-sold to women* [as] *a physiological way to deliver babies. When actually the only mammal that deliver under water are whales, and even they don't actually deliver under water… all the whales circle round and create a sort of bubble raft in order to make it more safe.* (N 2004)

*Even hippopotamuses come out of the water to deliver on land.* (O 2007)

One obstetrician noted a need for greater promotion of the benefits of pool use amongst medical staff.

*I think there needs to be a higher awareness of the benefits…* [They] *need to be much more clearly shown* [to] *obstetricians… you know they have heard some reports of aspiration and pneumonia, and of babies dying etc. And that is what sticks in their mind, they don't hear the rest of the stories where the birth went better.* (O 1004)

### Factors influencing birth pool use

Medical staff broadly identified the same issues related to pool accessibility as those reported by women and midwives. Pool availability was frequently cited as a potential barrier, although most interviewees stated there were sufficient pools to meet the perceived low demand for waterbirth in their own unit. Obstetricians saw waterproof CTG equipment as a useful tool, particularly in obstetric-led units.

*Previously I think it was only babies that didn't need to be monitored… could go in the pool and that would pretty much exclude the majority* [of] *people that were up here in the consultant led unit, but now that we've got the monitoring of babies there's a bit more inclusion criteria for those that want to… I think that's useful actually.* (O 1005)

Although not a factor mentioned by women and midwives, medical staff proposed that limited numbers of midwives with appropriate experience may constrain pool use. One neonatologist suggested births in water required a greater number of staff as they were more labour intensive in terms of filling the pool. Furthermore, in their unit the number of midwives allocated to pool births had increased due to concerns about evacuating women from the pool in an emergency.

As identified by midwives and women, medical staff concurred that maternity unit culture affected the extent to which pool use was supported, suggesting some units were over-cautious about waterbirth due to safety concerns.

*I do think there is still some resistance to* [waterbirth] *in the obstetric field. So better information* [about it] *wouldn't be a bad thing I think… I think people are worried about infections, people are worried about aspirations, pneumonia in babies. So yeah there are obstetricians who take a long time to change their views.* (O 1004)

As suggested by women, one obstetrician reflected that eligibility criteria for pool use could be arbitrary and dependent on individual staff opinion.

*To be honest I think* [eligibility criteria for pool use] *just comes down to which staff are on that day. So, yeah I mean there will be situations where the staff on would refuse to look after someone if they felt the risk was too high. But the same… woman might be supported by a different member of staff.* (O 1002)

Some obstetricians suggested there was a need for greater promotion of pool use by maternity unit staff, to ensure women are aware of this option. Medical staff identified that there was little proactive support for waterbirth in obstetric-led units.

*I think in the consultant antenatal clinics there is insufficient promotion of water for the women… in the MLU, you know, going in the water is just like accepted as a good normal option. I think once women become high risk they don't get that option promoted enough. So it may be that they could go in the water, but nobody has actually said,* [and] *lots of women don't ask.* (O 1003)

Some interviewees proposed there is little desire for waterbirth from women themselves. Medical staff suggested demand for waterbirth varies across the UK, with women wishing to use a pool tending to be well-educated and informed.

*I think the women that tend to want waterbirths… in my experience tend to be the more, perhaps more educated women… it's perhaps not as widely thought of in certain ethnic groups, social classes, and certain parts of the country.* (O 1008)

## Discussion

Our research identified factors influencing the use of birth pools in the UK through exploring the attitudes and experiences of key stakeholders. Overarching categories were resources, unit culture and guidelines, and staff endorsement. This is the first UK-based study to encompass the perspectives of women, midwives and medical staff. Findings highlight several changes to practice that could enable more women to gain access to a pool for labour and birth.

In terms of resources, availability of pools was clearly a substantial factor influencing the accessibility of waterbirth, as identified in previous research (Young and Kruske, 2012). Findings also provide new insights into the secondary effects of limited pool availability, such as the impact on midwives’ experience of and confidence in facilitating waterbirth. Although a lack of availability was reported by women and midwives, waterproof CTG equipment was seen as a useful tool that could increase the proportion of women able to access water immersion during labour on obstetric-led units. For example, more widespread availability and use of waterproof CTG equipment could provide women with an indication for continuous electronic fetal heart rate monitoring in labour with access to water immersion analgesia. We did not find evidence of overt blocking of pool rooms by senior midwives, as reported by Russell (2011, 2016). However, both women and midwives did identify instances where allocation of birthing rooms with pools meant women who wished to use a pool were unable to do so.

In respect of unit culture and guidelines, strict eligibility criteria for pool use (as found by Newnham et al., 2015, 2017) had a clear impact on the accessibility of waterbirth. In line with previous studies (e.g. Cooper, McCutcheon et al., 2017, in press), both women and medical staff suggested assessments of eligibility were subjective rather than evidence based. Furthermore, specific safety concerns raised by neonatologists in this study (such as water aspiration and infection) do not appear to be substantiated by available evidence (Young and Kruske, 2013).

Examination of the UK context highlighted differences between midwifery-led and obstetric-led maternity units. While the former were generally perceived as supportive of pool use, the latter were described as an over-medicalised environment in which pool use was seen as unusual, and therefore restricted. Exploring the perspectives of medical staff facilitated understanding of the reasons behind these differences in culture, building on previous quantitative research (Plint and Davies, 2016). Obstetricians and neonatologists acknowledged there was hypervigilance around waterbirth, attributing this to a lack of evidence on the safety of pool use and skewed perceptions of risk due to experiences being confined to complicated births.

Support for waterbirth from midwives and medical staff, as gatekeepers, was a key influence on pool use. Although women receiving midwifery-led care generally felt supported to have a pool birth, some resistance was reported. Midwives suggested this was due to a lack of confidence, arising from limited skills or experience in this area and a lack of support from senior staff. As found by Plint and Davies (2016), while midwives valued pool use as an option for women, medical staff tended to be sceptical about the potential benefits.

In support of Russell's (2016) findings, participants suggested waterbirth tended not to be actively offered as an option, therefore there was a reliance on women themselves requesting to use a pool. This is contrary to NICE (2014) guidance, which states that women should be offered the opportunity to use a pool during labour. A lack of promotion of waterbirth antenatally may substantially impact on pool use. For example, Baxter (2006) found in one UK birth centre that 89% of women who used a pool over three years had received information about pool use from their midwife prior to labour. Women in this study emphasised the need for information about waterbirth to be provided proactively during antenatal care.

Some medical staff suggested there is low demand for waterbirth amongst particular groups of women, proposing that those who wish to use a pool tend to be well-educated and from particular ethnic groups and social classes. Although this was not supported by discussion group findings, the majority of women taking part had given birth in water, therefore low desire for pool use was unlikely to be identified.

### Strengths and limitations

A strength of this study is the inclusion of medical staff alongside women and midwives. Examining the research issue from multiple perspectives enabled a comprehensive exploration of the factors influencing birth pool use. Comparing the attitudes and experiences of medical staff and midwives strengthened understanding of the differences in culture between midwifery-led and obstetric-led maternity units.

Conducting interviews rather than online discussion groups with medical staff may have affected responses. For example, the interactive nature of discussion groups can elicit views that would not be generated in individual interviews (Kidd and Parshall, 2000), while the anonymity afforded by online participation may mean participants are more comfortable in disclosing sensitive information or controversial views (Williams et al., 2012; Woodyatt et al., 2016). However, as found in previous research (Guest et al., 2017), interviews with medical staff in this study enabled the generation of a broad range of themes from a limited number of participants. Interviewees appeared to be confident to disclose potentially controversial views; therefore this method was felt to be suitable for the participant group.

A limitation of the research was that women participating in the discussion group were not representative in terms of pool use. As the vast majority had previously used a pool or bath during labour, there was little opportunity to explore the perspectives of those who were unable to use a pool or did not wish to do so. This could provide useful insights, for example into women's concerns about pool use, an area little explored in previous research. Furthermore, although utilising online discussion groups enabled access to a geographically diverse population, this method may have resulted in sampling bias through excluding those with low levels of digital literacy (Ferrante et al., 2016). To enhance security of the online groups, registration was a two-stage process, which may have been a barrier to participation. Technical difficulties in accessing the discussions were also reported by some participants, which is likely to have affected participation rates.

### Implications for practice

Access to birth pools could be improved through ensuring greater availability of pools and waterproof CTG equipment on maternity units, even where demand appears to be low. This would increase visibility and awareness of this option amongst women, and enable midwives to enhance their experience, skills and confidence in facilitating waterbirth.

Ensuring maternity unit guidelines and eligibility criteria for water immersion are evidence based and applied consistently could help ensure women are not unnecessarily blocked from using a pool. Furthermore, evidence-based information for medical staff on the potential benefits and relative risks of pool use, including case studies of normal births, may increase support for waterbirth. Interprofessional study days could also be valuable in enhancing midwives’ confidence and alleviating the concerns of medical staff.

It is important that information about pool use is provided antenatally and that water immersion is proactively offered when women are in labour, to ensure all women are aware of this option.

### Areas for future research

Further exploration of the differences in culture between obstetric-led and midwifery-led maternity units could strengthen understanding of how waterbirth is perceived and facilitated. This could encompass examination of unit polices, physical environment and the attitudes and experiences of staff and women, and the interactions between these factors. Stage two of this research will comprise an in-depth exploration of factors influencing the use of birth pools and waterbirth, through case studies of UK obstetric- and midwifery-led maternity units.

Current guidance (NICE, 2014) indicates there is ‘insufficient high-quality evidence to either support or discourage giving birth in water’. Therefore, there is a need for robust evidence on the relative risks and benefits of waterbirth, to inform practice and support women to make informed birth choices.

Research with women who did not use a pool during labour would provide a useful insight into the experiences, knowledge and beliefs of those who do not wish to use a pool or have little awareness of this option. This should include identification of any socio-demographic differences.

## Conclusion

Resources, maternity unit culture and guidelines, and staff endorsement are key factors influencing the use of birth pools in the UK. This study suggests maternity units could improve access to water immersion through increasing pool availability (even where there appears to be low demand for waterbirth), implementing evidence-based guidelines on pool use and ensuring awareness of the benefits and relative risks amongst medical staff. Midwives can play a key role in raising the profile of waterbirth, through providing information antenatally and proactively offering water immersion as an option to women in labour.

## Funding sources

This study is funded by the National Institute for Health Research (NIHR) HTA programme (project reference 16/149/01). The views expressed are those of the authors and not necessarily those of the NIHR or the Department of Health and Social Care.

## Ethical approval

The study has been approved by Wales Research Ethics Committee 3.

## Clinical trial registry and registration number

ISRCTN13315580

## Declaration of Competing Interest

The study is funded by the National Institute for Health Research (NIHR) HTA programme (project reference 16/149/01). The Royal College of Midwives make a financial contribution to support BH's appointment.
